# Spatiotemporal Distribution and Risk Factors of African Swine Fever Outbreak Cases in Uganda for the Period 2010–2023

**DOI:** 10.3390/v17070998

**Published:** 2025-07-16

**Authors:** Eddie M. Wampande, Robert Opio, Simon P. Angeki, Corrie Brown, Bonto Faburay, Rose O. Ademun, Kenneth Ssekatawa, David D. South, Charles Waiswa, Peter Waiswa

**Affiliations:** 1 College of Veterinary Medicine, Animal Resources and Biosecurity, Makerere University, Kampala P.O. Box 7062, Uganda; robertop4343@gmail.com (R.O.); angksimon7722@gmail.com (S.P.A.); charles.waiswa@mak.ac.ug (C.W.); peter.waiswa@mak.ac.ug (P.W.); 2 Ministry of Agriculture, Animal Industry and Fisheries, Entebbe P.O. Box 102, Uganda; ademunrose@yahoo.co.uk; 3 Lifestock International, Athens, GA 30606, USA; corrie@lifestock.org; 4 Foreign Animal Disease Diagnostic Laboratory, National Veterinary Services Laboratories, National Bio and Agro-Defense Facility, United States Department of Agriculture, Manhattan, KS 66502, USA; bonto.faburay@usda.gov; 5 Department of Science, Technical and Vocational Education, Makerere University, Kampala P.O. Box 7062, Uganda; kssekatee@gmail.com; 6 United States Department of Agriculture, George Washington Carver Center, 5601 Sunnyside Avenue, Beltsville, MD 20705, USA; david.d.south@usda.gov

**Keywords:** African Swine Fever (ASF), Uganda, spatiotemporal trends, ASF clusters

## Abstract

This paper describes the spatiotemporal distribution and risk factors of African Swine Fever (ASF) in Uganda for the period of 2010 through 2023. The study utilized a comprehensive dataset from monthly reports (2010–2023) from District Veterinary Officers (DVOs), the Ministry of Agriculture, Animal Industry and Fisheries (MAAIF), and the Food and Agriculture Organization, Uganda. Using GPS coordinates, ASF cases were mapped using QGIS to show ASF distribution and spread in Uganda. Moran’s I analysis was used to delineate clusters of ASF. A total of 1521 ASF cases were recorded. The data show that cases of ASF were disseminated throughout the country, with more cases of ASF documented in the central region and border districts (hotspots for ASF), and few cases were reported in Acholi, Karamoja, and Lango, Ankole, West Nile, and Kigezi sub-regions. The time series analysis revealed incidences of ASF disease occurring year-round; notable peak cases were observed in some districts, and districts with ≥30,000 pigs reported higher cases of ASF. The Moran’s I (≥1) analysis showed that ASF is either aggregated (*p* = 0.01), especially in central districts bordering Tanzania and lake shores, or sporadic in occurrence. The disease was present in 66% of the districts, with ASF occurring throughout the year. More cases were aggregated in central and border districts and districts with large pig populations (≥30,000). Sporadic cases were reported in districts bordering the DRC, Sudan, Kenya, the lake shores, Karamoja, Acholi, and Lango sub-regions.

## 1. Introduction

African Swine Fever (ASF) is a contagious, highly fatal hemorrhagic disease of domestic pigs, caused by a double-stranded DNA African Swine Fever Virus (ASFV) of the family Asfarviridae [1]. The disease has been recognized for over a century, with the first report published in 1921 [2], and now assumes a global spread with outbreaks being detected outside African countries [3,4], which affirms the threat ASF poses to the pig production sector worldwide. The persistence of the disease is a result of its complexity in the epidemiology of transmission, which involves the domestic cycle (pig to pig or fomite to pig) and the sylvatic cycle (tick to domestic pig or wildlife to tick) [5], the lack of an effective vaccine, and the preponderance of raising by smallholders, often with insufficient ability to implement biosecurity. In Uganda, ASF is considered to be an endemic disease with pig–pig or fomites as the main mode of transmission [5,6]. The perpetuation of the disease in wildlife is dependent on soft ticks due to the lack of horizontal and vertical transmission between warthogs [7]. The disease in domestic pigs can present in an acute form, where mortalities can approach 100%, or a sub-acute/chronic form depending on the virulence of the infecting genotype of ASFV [4,8]. The occurrence and spread of the disease is associated with high economic losses due to pig deaths and the human resources required to impose quarantine. Nevertheless, in Uganda, the actual economic losses of ASF outbreaks are underreported due to a general lack of record keeping by smallholder farmers [9,10]. The absence of an effective vaccine or drug against ASFV compounded with poor biosecurity measures and the persistence of a wildlife reservoir all make ASF control and eradication a formidable challenge. Moreover, halting disease spread requires tools for the early detection of outbreaks and stringent sanitary measures as well as knowledge of soft tick and wildlife epidemiology.

Studies elsewhere have shown that ASF outbreaks can occur nearby or at some distance apart, and the time interval between cases may vary [8,11,12]. The place and time of the occurrence of ASF can depend on the physical features of the affected area, the geographic distribution of the domestic and wild animal populations at risk, the frequency of contact between them, and the presence of the virus [8]. Understanding ASF dynamics requires knowledge of potential sources of infection/reservoirs, when it is likely to strike, transmission mechanisms, and risk factors. Knowing the spatiotemporal distribution and risk factors associated with the spread of disease will provide insights into the current scenario of ASF in Uganda, and help to target subsequent areas for possible outbreaks. The spatiotemporal analysis method, based on risk maps of the outbreaks, is valuable for understanding its spread patterns and to identify high-risk areas for the application of targeted control measures [13,14,15]. For instance, spatial autocorrelation analysis can identify whether the overall distribution of disease is clustered or dispersed and detect the location of clusters and outliers [16,17]. Spatiotemporal scanning statistical analysis can detect high-risk clusters of disease in continuous time and space [17,18]. Knowing the hotspots of ASF disease and the probable time of occurrence provides a method for describing the confines of surveillance and predicting the point when and where the next outbreak is likely to occur [8,19]. Such information aids in the advancement of risk assessment and mitigation strategies with the ultimate goal of preventing future occurrences of the disease in the country/region [20]. To our knowledge, the spatiotemporal distribution patterns of the ASF in Uganda are poorly understood and worthy of further exploration. An extensive abattoir-based study by Okwasiimire et al. [21] was limited by sampling from central Uganda only. A second study by Atuhaire et al. [22] included pigs from the entire country, but the sample size was limited, making it difficult to draw extrapolations. The aim of this study was to use a very large sample size (1521 positive outbreaks) from the entire country, covering 13 years.

## 2. Materials and Methods

### 2.1. Data Source and Collection

Retrospective data on ASF outbreaks (1210 cases) in Uganda during 2010–2022 were retrieved from the Ministry of Agriculture Animal Industry and Fisheries (MAAIF) and the Food Agriculture Organization (FAO), Uganda archives. Prospective data (cases 311) on ASF outbreaks in Uganda during 2023 were obtained from the Central Diagnostic Lab (CDL) at the College of Veterinary Medicine, Animal Resources and Biosecurity. Additional information captured included the name of the village and district where the sample was collected, the ASF outbreak reported date, the date of sample collection, the location (latitude and longitude) of the outbreaks, and the laboratory results (all cases were confirmed by PCR). An outbreak was defined as one or more ASF cases in the village/district confirmed by PCR.

### 2.2. Geographical Spatial Analysis

The retrospective and prospective case data were combined for each district and mapped using QGIS, an open-source geographic information system (GIS) software. The projected coordinate system used was WGS 84/UTM zone 34 N (EPSG:32634). Uganda’s administrative borders were from the Esri Living Atlas. Data were displayed/symbolized by district in a choropleth map, with gray indicating zero cases and darker colors indicating more cases.

### 2.3. Time Series and Cluster Analysis

The retrospective and prospective data were plotted on a bar graph per quarter, considering the number of outbreak cases per month from 2010 to 2023, which defined the occurrence of the disease per year. The absolute number of ASF disease cases was plotted against months (quarterly). The cluster analysis statistic test was performed using Moran’s I. According to Moran’s I software, retrospective, and prospective data were fed into a GIS tool to layer the outbreak cases onto the map of Uganda. The global positioning system (GPS) was then transformed into shape files, which were then used to develop the raster images that were presented in different colors on the map of Uganda. The Moran’s I statistic ranged from −0.3 to 0.3, where a positive value indicated a positive spatial autocorrelation or clustering (similar values tend to occur near each other) and a negative value indicated a negative spatial autocorrelation or dispersion (dissimilar values tend not to occur near each other). A value close to 0 suggested no significant spatial autocorrelation (random spatial pattern).

## 3. Results

### 3.1. Geographical Distribution of ASF Outbreak Cases by District for the Period 2010–2023

**Figure 1:**
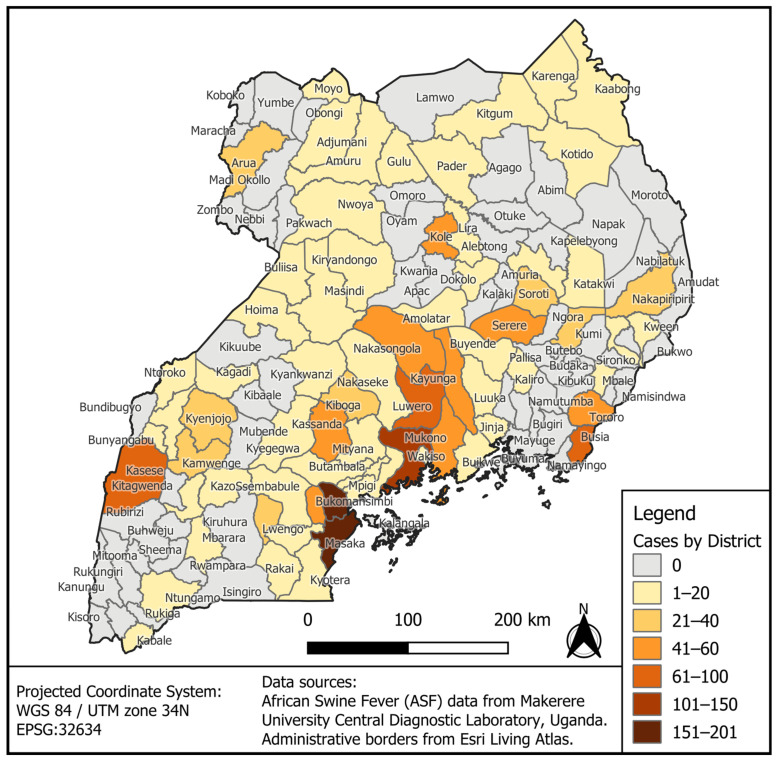
A choropleth map showing the spread of ASF in Uganda from 2010–2023. The GIS position for each ASF outbreak case was mapped onto the map of Uganda in QGIS to show the layering of the disease across the Ugandan districts.

The GPS coordinates for places of reported outbreak cases of African Swine Fever disease (ASFD) were layered on the map of Uganda. The data show that the disease was disseminated nationwide, with 55% of the districts affected from 2010 to 2023, with variable disparities (Figure 1 and Table A1). The districts situated on the eastern (Masaka, Bukomansimbi, Kalungu) and northern side (Kampala and Wakiso) of Lake Victoria had the highest number (100–200 cases) of ASF outbreak cases. Districts in central Uganda (Mukono, Kayunga, Nakasongola, Kabasanda, and Bukomansimbi) and border districts such as Kasese, Nakapiripiriti, Tororo, and Busia had ASF cases ranging between 40 and 100. Such geographical areas may represent areas with a high risk of ASF occurrence. The districts in the Acholi sub-region and Karamoja region had comparatively lower outbreak numbers (1–60 cases). In the districts of Lamwo, Napak, Abim, Oyam, Otuke, Omoro, Moroto, and Agago (Lango and Karamoja region), no single case was reported. The districts of central Uganda and areas surrounding Lake Victoria and the eastern and western borders had the highest number of ASF cases.

### 3.2. Pig Number Estimates vis-à-vis ASF Outbreaks in Uganda

**Figure A1:**
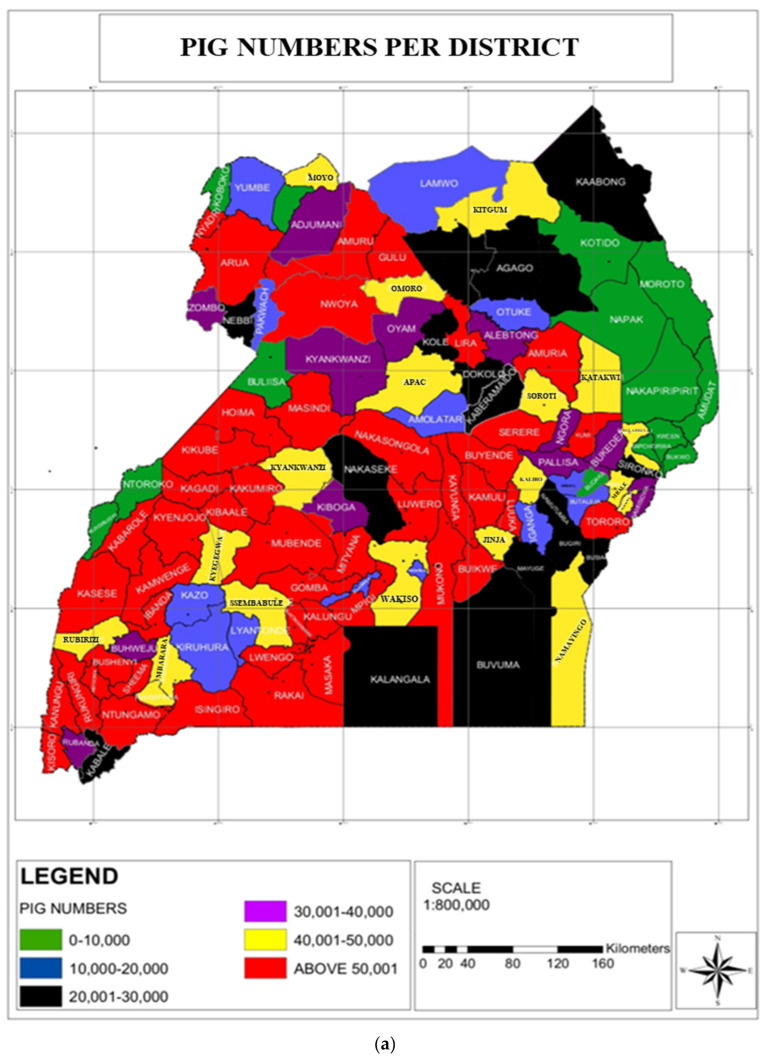

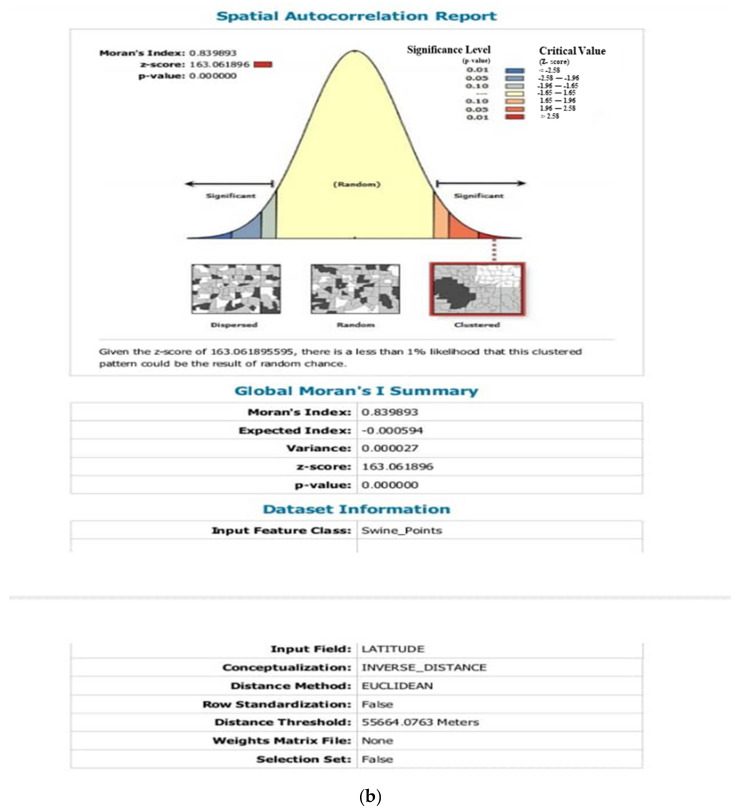
Supplementary data showing pig numbers per district in Uganda as per data from 2023 UBOS Livestock census (**a**), and the autocorrelation report (**b**) showing the statistical analysis delineating disease occurrence from 2010 to 2023.

Domestic pigs are reared throughout the country; the majority of the pigs (above 30,000 pigs/district) are found in Central and Western districts of Uganda, interspersed with districts with the number of pigs ranging between 10,000 and 20,000. Northeastern districts have the lowest number of pigs, with fewer than 10,000 (Figure A1). Notably, the number of ASF outbreak cases was significantly higher in districts with a high number of domestic pigs than in places with fewer or no pigs at all.

### 3.3. Time Series Distribution of ASF Cases by Month from 2010 to 2023

**Figure 2:**
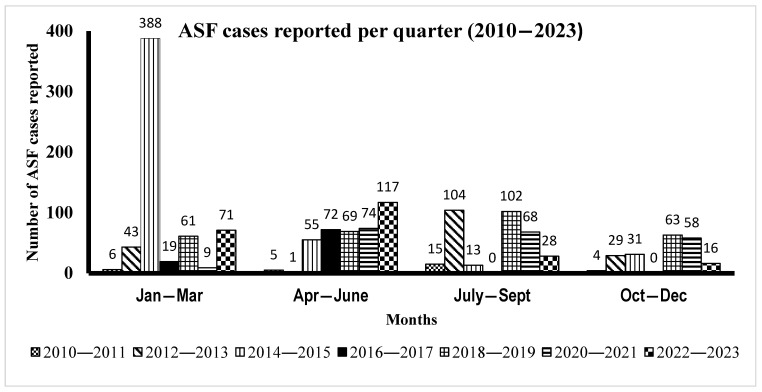
Yearly occurrence of ASF cases for the period of 2010 through 2023: Reported cases of ASF were plotted against the month when the case(s) were reported on a quarterly basis for a given year.

The temporal distribution analysis of ASF outbreak cases from 2010 to 2023 (Figure 2) demonstrated the occurrence of disease throughout the year, with cases being reported every month and no observed pattern of ASF outbreak occurrence. Overall, the occurrence of ASF disease outbreaks varied significantly across different months and years. For instance, from 2010 to 2011, the number of cases ranged between 4 and 15, with the highest number of detected outbreaks (15 cases) reported between July and September. The period of 2012–2013 had outbreak cases ranging from 43 to 104, with a substantial peak (104 outbreaks) during July–September. From 2014 to 2015, outbreak cases ranged from 31 to 388, marked by an unprecedented outbreak in January–March, with 388 cases reported. The period of 2016–2017 had between 19 and 72 cases; no cases were reported in the third and fourth quarters (July–December). The years of 2018 to 2019 witnessed a relatively high number of cases (61–102) spread more evenly throughout the year, peaking with 102 cases in July–September. From 2020 to 2021, ASF outbreak cases ranged between 9 and 74, fairly evenly distributed, with the highest number of cases (74) observed between April and June. Between 2022 and 2023, 16 to 117 outbreaks were confirmed, with the highest number of cases (117) recorded between April and June. Overall, the disease occurred throughout the years, with April–September (two quarters) showing the highest number of ASF outbreaks for the period 2010–2023.

### 3.4. Analysis of ASF Disease Cluster

**Figure 3:**
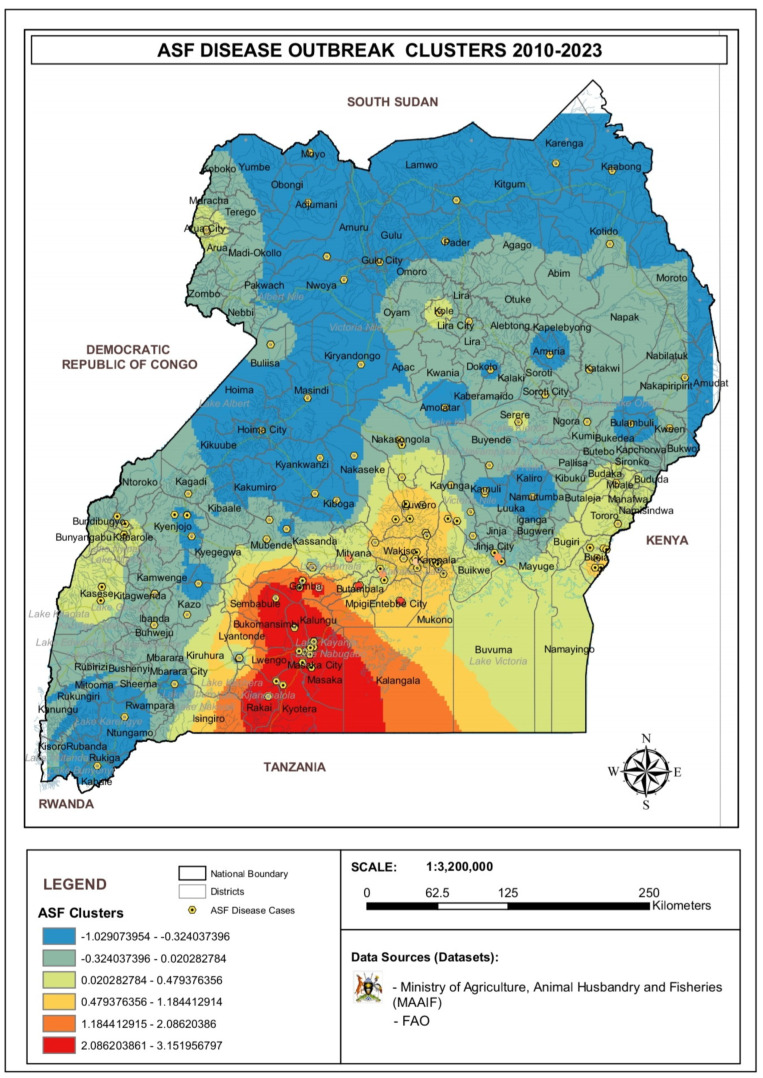
Clustering and sporadic spread of African swine fever outbreaks observed from 2010 to 2023: The Moran’s I-defined spatial aggregation of ASF in the central Ugandan district bordering Tanzania and sporadic spread in the rest of the districts.

**Figure A2:**
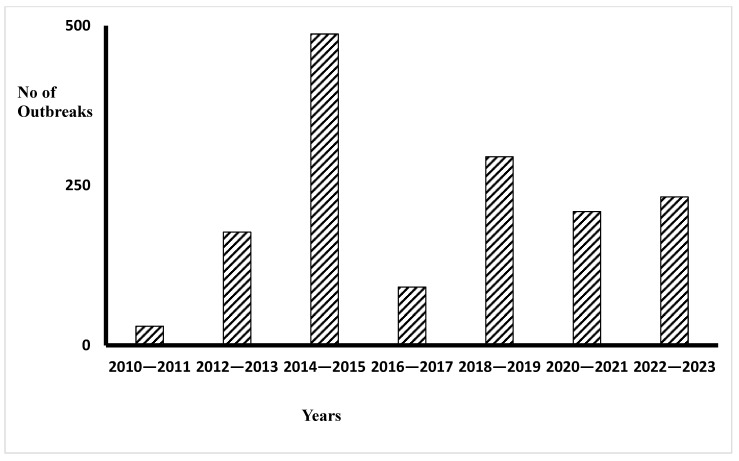
Supplementary figure showing annual description of ASF outbreak cases for the period 2010—2023.

The Moran’s I analysis classified the ASF disease occurrence in Uganda into six categories, with groups having observed Moran’s I values greater or less than 1. A significant cluster (*p* = 0.01) was observed in the South-Central districts bordering Tanzania, confirming a hotspot for ASF disease occurrence, while for the rest of the districts in Uganda, a dispersal or random mode of distribution of ASF outbreak was detected (Figure 3 and Figure A2). Randomly distributed cases of ASF disease outbreaks were mapped along the border districts of western Uganda, along the shores of Lake Kyoga, West Nile, and eastern regions as well as Acholi, Karamoja, and Lango sub-regions. The distribution of cases was either clustered, which may indicate a common source of infection, or randomly dispersed, where infection can be acquired through different routes.

## 4. Discussion

In this study, we developed a risk map to provide an indication of the spatial distribution of ASF risk using 1521 ASF-recorded outbreaks from 2010 to 2023. Our findings show that a minimum of 55% (74/135) of the districts in Uganda have reported at least one case of ASF throughout the years. In addition, the raster map demonstrates the disease occurring in aggregations, especially in central Uganda and the Tanzanian–Ugandan border or sporadic cases reported along the Ugandan border districts of Kenya, South Sudan, the Democratic Republic of Congo (RDC), and Rwanda. Lastly, international border districts and high pig populations are the major risk factors for the disease. Overall, the disease is endemic in most districts of Uganda, highlighting geographical hotspots, denoting high densities of the disease and fluctuating disease trends occurring throughout the years.

Our data demonstrate that ASF is endemic in most districts in Uganda, with the majority of the outbreak cases seen in the central regional districts (Kampala, Wakiso, Luwero, Masaka, and Bukomansimbi), along the shores of Lake Victoria, on the Uganda–Tanzania border, and in eastern Uganda [23,24]. Such districts experienced a disproportionately high number of ASF cases, making the region a hotspot for the disease. The 2024 livestock census reveals that these regions also had high pig and human populations, with several districts having more than 50,000 pigs. Such high pig densities are critical factors in the rapid spread of ASF [25], as the disease can easily be transmitted through direct contact (pig–pig), contaminated feed, and materials used in pig husbandry [26]; yet, the human population increases the demand for pork consumption. Additionally, the proximity of these districts to Kampala—a major pig trade hub for live pigs [21] due to its economic strength, increased demand for pork because of a high population, and festivals can increase the risk of the introduction and spread of ASF in these areas. The appearance of the disease at international border districts may imply informal movement of infected pigs and pork products from neighboring countries that will join the marketing chain, thus importing the disease into the respective districts. Such a scenario is possible: studies elsewhere have described a genetic relatedness of ASFV circulating within Uganda and Kenya, highlighting the significance of virus transmission between the two countries [27]. The widespread nature of ASF in most districts in the country could be attributed to the poor implementation of government policies on disease control, for instance, the adoption of quarantine measures in cases of ASF outbreak; the control of the movement of pigs where, in some cases, animals move without a health permit or pig movement permits; and poor knowledge of the biosecurity measures in the control of ASF [25,28]. Overall, such districts are geographical areas with a potentially high risk of ASF prevalence and, thus, should be targeted for disease control. Furthermore, border districts like Tororo, Busia, Kasese, Kyotera, and Arua reported a relatively high number of ASF outbreaks. These areas are susceptible to international border transmission of ASF due to trade involving live pigs and pork between neighboring countries [25,29], in case biosecurity is compromised [28,30]. On the other hand, the northeastern part of Uganda, including the Lango region, Acholi sub-region, and Karamoja region (Napak, Otuke, Moroto, and Agago), recorded no cases of ASF during the study period. The lower pig density and geographical isolation (poor road networks) that do not allow pig–pig contact in these regions likely contributed to the absence of ASF [12]. In addition, in this study, we set out to describe the spatial autocorrelation of ASF. Our findings demonstrate the potential infection and spread risk zones of ASF in Uganda, with disease aggregation being observed in central Uganda and along the lake shores. Such a pattern points to localized transmission within districts. The disease clustering of ASF cases within certain regions, as highlighted by Moran’s I statistical tests, indicates point-source outbreaks arising from a common source (cluster) [31,32] etiology that might be due to related or unrelated strains, although in Uganda, only strain IX of ASFV has been reported [33]. The probable cause of this could be the high population density of domestic pigs in the central region that promotes pig–pig transmission; the intensive pig management enterprise, which is a common practice in the center; or socioeconomic factors that provide the purchase power and costs required in the management of the pig enterprise, which is a strength of the population in the central region [34,35,36]. Additionally, we also observed a sporadic spread of ASF in some of the Ugandan borders and inland districts. This randomness of the spread of ASF is probably due to, firstly, the sparse pig population in the districts, which minimizes pig–pig (infected pig versus susceptible pig) contact to drive transmission, and secondly, the underreporting of cases due to the lack of laboratory infrastructure and technical knowledge to detect disease [37,38]. Such geographical environments are potential sources of infections for future outbreaks, so they should be targeted for localized intervention, such as stricter biosecurity measures and movement restrictions to prevent ASF from spreading further [39,40].

Furthermore, in this paper, we described the relationship between time (months) and ASF occurrence (outbreak) in Uganda. The data show no particular trend/pattern of occurrence of the disease; nonetheless, at least one case of ASF has been reported every month (January–December) or quarter of the year for the study period, without any discernible seasonality or quarterly outbreak pattern seen in the district. Studies on rainfall and temperature distribution in Uganda at diverse spatiotemporal measures showed insignificant variation annually [41,42]. Yet rainfall and temperature (16–40 °C) have substantial effects on ASF outbreaks, because they promote the survival of the ASFV in the environment and the vector (soft tick), thus perpetuating transmission throughout the year [24,43,44]. Moreover, high incidences of the outbreak were observed between April and September; this period corresponds with a period with high rainfall, and such an ecosystem favors the survival of vectors (soft ticks) that are transmitters of ASFV [24,25]. From our previous data, we demonstrated the exposure of pigs to soft tick bites [45]. Notwithstanding, we observed that, in some years, there were periods with an exceedingly high number of outbreaks. We speculate that there was probably a lack of consistency in reporting due to inadequate resources, restocking with infected pigs in districts, and the burden of quarantine. With that background, the lack of a well-defined seasonal or quarterly trend of ASF occurrence provides insight that ASF control strategies must be continuous rather than limited to certain times of the year.

## 5. Conclusions

Our findings describe a nationwide distribution of ASF with at least 66% of the districts affected by the disease, with the central regional and cross-border districts recording the highest number of cases; thus, targeted interventions such as ASF continuous surveillance and preventive measures through the inspection of pigs and their products before entry at cross-border areas could significantly reduce the overall incidence of the disease. Moreover, ASF outbreaks are seen occurring throughout the year (January–December), and such findings underscore the need for continuous surveillance and preparedness to promptly confirm the disease and prevent its spread to many other places. In addition, high pig populations, pig trade movement activities, and border districts are major risk factors. Therefore, mitigating the disease requires strict attention to these factors.

## 6. Limitation

This study only dealt with severe cases of ASF in Uganda, excluding the sub-clinical cases (carrier or chronic cases) that make up a small percentage of the true numbers of active disease. Furthermore, this report only describes incident cases of ASF, which do not reflect the true burden of ASF in Uganda. Thus, we cannot rule out the fact that the outbreaks were underreported, since most farmers sell off their pigs whenever there are suspected cases in the vicinity for fear of the restriction of pig movements (quarantine), even without confirming the cause of the death of the pig.

## Data Availability

All data generated by this study have been submitted along with this manuscript in the form of Appendix A, Appendix B and Appendix C.

## Abbreviations

The following abbreviations are used in this manuscript:

ASF	African Swine Fever
ASFV	African Swine Fever Virus
DRC	Democratic Republic of Congo
MAAIF	Ministry of Agriculture, Animal Industry and Fisheries
FAO	Food Agriculture Organization
CDL	Central Diagnostic Lab

## Appendix C

**Table A1:** Disease cases per year in the Ugandan districts with quarterly stratification.

Year	January–March	April–June	July–September	October–December	Total
2010–2011	6	5	15	4	30
2012–2013	43	1	104	29	177
2014–2015	388	55	13	31	487
2016–2017	19	72	0	0	91
2018–2019	61	69	102	63	295
2020–2021	9	74	68	58	209
2022–2023	71	117	28	16	232

## References

[1] Alonso C., Borca M., Dixon L., Revilla Y., Rodriguez F., Escribano J.M. (2018). ICTV Report Consortium. ICTV Virus Taxonomy Profile: Asfarviridae. J. Gen. Virol..

[2] Eustace Montgomery R. (1921). On A Form of Swine Fever Occurring in British East Africa (Kenya Colony). J. Comp. Pathol. Ther..

[3] Chapman D.A.G., Darby A.C., Da Silva M., Upton C., Radford A.D., Dixon L.K. (2011). Genomic Analysis of Highly Virulent Georgia 2007/1 Isolate of African Swine Fever Virus. Emerg. Infect. Dis..

[4] Li Z., Chen W., Qiu Z., Li Y., Fan J., Wu K., Li X., Zhao M., Ding H., Fan S. (2022). African Swine Fever Virus: A Review. Life.

[5] Penrith M.-L., Vosloo W., Jori F., Bastos A.D.S. (2013). African swine fever virus eradication in Africa. Virus Res..

[6] Muwonge A., Munang’andu H.M., Kankya C., Biffa D., Oura C., Skjerve E., Oloya J. (2012). African swine fever among slaughter pigs in Mubende district, Uganda. Trop. Anim. Health Prod..

[7] Jori F., Bastos A.D.S. (2009). Role of Wild Suids in the Epidemiology of African Swine Fever. EcoHealth.

[8] Korennoy F.I., Gulenkin V.M., Malone J.B., Mores C.N., Dudnikov S.A., Stevenson M.A. (2014). Spatio-temporal modeling of the African swine fever epidemic in the Russian Federation, 2007–2012. Spat. Spatio-Temporal Epidemiol..

[9] Chenais E., Boqvist S., Emanuelson U., Von Brömssen C., Ouma E., Aliro T., Masembe C., Ståhl K., Sternberg-Lewerin S. (2017). Quantitative assessment of social and economic impact of African swine fever outbreaks in northern Uganda. Prev. Vet. Med..

[10] Aliro T., Odongo W., Ståhl K., Dione M.M., Okello D.M., Masembe C., Chenais E. (2023). Actions and perceived impact of African swine fever control measures along the smallholder pig value chain in Uganda. Trop. Anim. Health Prod..

[11] Gulenkin V.M., Korennoy F.I., Karaulov A.K., Dudnikov S.A. (2011). Cartographical analysis of African swine fever outbreaks in the territory of the Russian Federation and computer modeling of the basic reproduction ratio. Prev. Vet. Med..

[12] Oganesyan A.S., Petrova O.N., Korennoy F.I., Bardina N.S., Gogin A.E., Dudnikov S.A. (2013). African swine fever in the Russian Federation: Spatio-temporal analysis and epidemiological overview. Virus Res..

[13] Perez A., AlKhamis M., Carlsson U., Brito B., Carrasco-Medanic R., Whedbee Z., Willeberg P. (2011). Global animal disease surveillance. Spat. Spatio-Temporal Epidemiol..

[14] Kimi R., Beegum M., Nandi S., Dubal Z.B., Sinha D.K., Singh B.R., Vinodhkumar O.R. (2024). Spatio-temporal dynamics and distributional trend analysis of African swine fever outbreaks (2020–2021) in North-East India. Trop. Anim. Health Prod..

[15] Ma J., Chen H., Gao X., Xiao J., Wang H. (2020). African swine fever emerging in China: Distribution characteristics and high-risk areas. Prev. Vet. Med..

[16] Franch-Pardo I., Napoletano B.M., Rosete-Verges F., Billa L. (2020). Spatial analysis and GIS in the study of COVID-19. A review. Sci. Total Environ..

[17] Shao Q., Li R., Han Y., Han D., Qiu J. (2022). Temporal and Spatial Evolution of the African Swine Fever Epidemic in Vietnam. Int. J. Environ. Res. Public Health.

[18] Ekakoro J.E., Lubega A., Kayaga E.B., Ndoboli D., Bluhm A.P., Wampande E.M., Blackburn J.K., Havas K.A., Norris M.H. (2022). An Investigation of Burkholderia pseudomallei Seroprevalence in Market Pigs Slaughtered at Selected Pig Abattoirs in Uganda. Pathogens.

[19] Adedeji A.J., Atai R.B., Gyang H.E., Gambo P., Habib M.A., Weka R., Muwanika V.B., Masembe C., Luka P.D. (2022). Live pig markets are hotspots for spread of African swine fever virus in Nigeria. Transbound. Emerg. Dis..

[20] Qiu J., Li R., Xiao Y., Xia J., Zhu H., Niu Y., Huang D., Shao Q., Cui Y., Wang Y. (2019). Spatiotemporal Heterogeneity in Human Schistosoma japonicum Infection at Village Level in Hubei Province, China. Int. J. Environ. Res. Public Health.

[21] Okwasiimire R., Kayaga E.B., Ekakoro J.E., Ndoboli D., Schumann K., Faburay B., Nassali A., Hauser C., Ochoa K., Wampande E.M. (2023). Spatiotemporal description of African swine fever virus nucleic acid and antibodies detected in pigs sampled at abattoirs in the greater Kampala metropolitan area, Uganda from May 2021 through June 2022. Porc. Health Manag..

[22] Kalenzi Atuhaire D., Ochwo S., Afayoa M., Norbert Mwiine F., Kokas I., Arinaitwe E., Ademun-Okurut R.A., Boniface Okuni J., Nanteza A., Ayebazibwe C. (2013). Epidemiological Overview of African Swine Fever in Uganda (2001–2012). J. Vet. Med..

[23] Ogweng P., Bowden C.F., Smyser T.J., Muwanika V.B., Piaggio A.J., Masembe C. (2024). Ancestry and genome-wide association study of domestic pigs that survive African swine fever in Uganda. Trop. Anim. Health Prod..

[24] Ungur A., Cazan C.D., Panait L.-C., Coroian M., Cătoi C. (2022). What Is the Real Influence of Climatic and Environmental Factors in the Outbreaks of African Swine Fever?. Animals.

[25] Costard S., Wieland B., De Glanville W., Jori F., Rowlands R., Vosloo W., Roger F., Pfeiffer D.U., Dixon L.K. (2009). African swine fever: How can global spread be prevented?. Philos. Trans. R. Soc. B Biol. Sci..

[26] Penrith M.-L., Vosloo W. (2009). Review of African swine fever: Transmission, spread and control. J. S. Afr. Vet. Assoc..

[27] Gallardo C., Ademun A.R., Nieto R., Nantima N., Arias M., Martín E., Pelayo V., Bishop R.P. (2011). Genotyping of African swine fever virus (ASFV) isolates associated with disease outbreaks in Uganda in 2007. Afr. J. Biotechnol..

[28] Ekakoro J.E., Nawatti M., Singler D.F., Ochoa K., Kizza R., Ndoboli D., Ndumu D.B., Wampande E.M., Havas K.A. (2023). A survey of biosecurity practices of pig farmers in selected districts affected by African swine fever in Uganda. Front. Vet. Sci..

[29] Chenais E., Lewerin S.S., Boqvist S., Ståhl K., Alike S., Nokorach B., Emanuelson U. (2019). Smallholders’ perceptions on biosecurity and disease control in relation to African swine fever in an endemically infected area in Northern Uganda. BMC Vet. Res..

[30] Wartenberg D. (2001). Investigating disease clusters: Why, when and how?. J. R. Stat. Soc. Ser. A Stat. Soc..

[31] Elliott P., Wakefield J. (2001). Disease clusters: Should they be investigated, and, if so, when and how?. J. R. Stat. Soc. Ser. A Stat. Soc..

[32] Okwasiimire R., Flint J.F., Kayaga E.B., Lakin S., Pierce J., Barrette R.W., Faburay B., Ndoboli D., Ekakoro J.E., Wampande E.M. (2023). Whole Genome Sequencing Shows that African Swine Fever Virus Genotype IX Is Still Circulating in Domestic Pigs in All Regions of Uganda. Pathogens.

[33] Aliro T., Chenais E., Odongo W., Okello D.M., Masembe C., Ståhl K. (2022). Prevention and Control of African Swine Fever in the Smallholder Pig Value Chain in Northern Uganda: Thematic Analysis of Stakeholders’ Perceptions. Front. Vet. Sci..

[34] Lichoti J.K., Davies J., Kitala P.M., Githigia S.M., Okoth E., Maru Y., Bukachi S.A., Bishop R.P. (2016). Social network analysis provides insights into African swine fever epidemiology. Prev. Vet. Med..

[35] Nantima N., Ocaido M., Ouma E., Davies J., Dione M., Okoth E., Mugisha A., Bishop R. (2015). Risk factors associated with occurrence of African swine fever outbreaks in smallholder pig farms in four districts along the Uganda-Kenya border. Trop. Anim. Health Prod..

[36] Chenais E., Sternberg-Lewerin S., Boqvist S., Emanuelson U., Aliro T., Tejler E., Cocca G., Masembe C., Ståhl K. (2015). African Swine Fever in Uganda: Qualitative Evaluation of Three Surveillance Methods with Implications for Other Resource-Poor Settings. Front. Vet. Sci..

[37] Penrith M.-L., Van Emmenes J., Hakizimana J.N., Heath L., Kabuuka T., Misinzo G., Odoom T., Wade A., Zerbo H.L., Luka P.D. (2024). African Swine Fever Diagnosis in Africa: Challenges and Opportunities. Pathogens.

[38] Mutua F., Dione M. (2021). The Context of Application of Biosecurity for Control of African Swine Fever in Smallholder Pig Systems: Current Gaps and Recommendations. Front. Vet. Sci..

[39] Dione M.M., Dohoo I., Ndiwa N., Poole J., Ouma E., Amia W.C., Wieland B. (2020). Impact of participatory training of smallholder pig farmers on knowledge, attitudes and practices regarding biosecurity for the control of African swine fever in Uganda. Transbound. Emerg. Dis..

[40] Mwaura F.M., Okoboi G. (2014). Climate Variability and Crop Production in Uganda. J. Sustain. Dev..

[41] Kilama Luwa J., Majaliwa J.-G.M., Bamutaze Y., Kabenge I., Pilesjo P., Oriangi G., Bagula Mukengere E. (2021). Variabilities and Trends of Rainfall, Temperature, and River Flow in Sipi Sub-Catchment on the Slopes of Mt. Elgon, Uganda. Water.

[42] Vial L. (2009). Biological and ecological characteristics of soft ticks (Ixodida: Argasidae) and their impact for predicting tick and associated disease distribution. Parasite.

[43] Mazur-Panasiuk N., Żmudzki J., Woźniakowski G. (2019). African swine fever virus—Persistence in different environmental conditions and the possibility of its indirect transmission. J. Vet. Res..

[44] Gallardo C., Okoth E., Pelayo V., Anchuelo R., Martín E., Simón A., Llorente A., Nieto R., Soler A., Martín R. (2011). African swine fever viruses with two different genotypes, both of which occur in domestic pigs, are associated with ticks and adult warthogs, respectively, at a single geographical site. J. Gen. Virol..

[45] Kayaga E.B., Wampande E.M., Ekakoro J.E., Okwasiimire R., Nassali A., Ochoa K., Hauser C., Ndoboli D., Havas K.A. (2024). Detection of antibodies against Ornithodoros moubata salivary antigens and their association with detection of African swine fever virus in pigs slaughtered in central Uganda. Front. Vet. Sci..

